# Displacement of the Greater Tuberosity in Humeral Head Fractures Does Not only Depend on Rotator Cuff Status

**DOI:** 10.3390/jcm10184136

**Published:** 2021-09-14

**Authors:** Lisa Klute, Christian Pfeifer, Isabella Weiss, Agnes Mayr, Volker Alt, Maximilian Kerschbaum

**Affiliations:** Clinic of Trauma Surgery, University Medical Center Regensburg, Franz-Josef-Strauss-Allee 11, 93053 Regensburg, Germany; christian.pfeifer@ukr.de (C.P.); weiss.isabella@t-online.de (I.W.); agnes-mayr@t-online.de (A.M.); volker.alt@ukr.de (V.A.); maximilian.kerschbaum@ukr.de (M.K.)

**Keywords:** humeral head fracture, greater tuberosity, rotator cuff, fatty degeneration

## Abstract

It is assumed that dorsocranial displacement of the greater tuberosity in humeral head fractures is caused by rotator cuff traction. The purpose of this study was to investigate the association between rotator cuff status and displacement characteristics of the greater tuberosity in four-part humeral head fractures. Computed tomography scans of 121 patients with Neer type 4 fractures were analyzed. Fatty infiltration of the supra- and infraspinatus muscles was classified according to Goutallier. Position determination of the greater tuberosity fragment was performed in both coronary and axial planes to assess the extent of dorsocranial displacement. Considering non-varus displaced fractures, the extent of the dorsocranial displacement was significantly higher in patients with mostly inconspicuous posterosuperior rotator cuff status compared to advanced fatty degenerated cuffs (cranial displacement: Goutallier 0–1: 6.4 mm ± 4.6 mm vs. Goutallier 2–4: 4.2 mm ± 3.5 mm, *p* = 0.020; dorsal displacement: Goutallier 0–1: 28.4° ± 32.3° vs. Goutallier 2–4: 13.1° ± 16.1°, *p* = 0.010). In varus displaced humeral head fractures, no correlation between the displacement of the greater tuberosity and the condition of the posterosuperior rotator cuff could be detected (*p* ≥ 0.05). The commonly accepted theory of greater tuberosity displacement in humeral head fractures by rotator cuff traction cannot be applied to all fracture types.

## 1. Introduction

In 1970, Neer established a new classification system for proximal humeral fractures that is still widely used in clinical practice today [1]. This classification system is based on the four main fracture fragments (humeral shaft, calotte, and greater and lesser tuberosity), firstly described by Codman in 1934 [2]. Neer assumed that traction of the rotator cuff is responsible for the characteristic fragment displacement, especially of the greater tuberosity. The supraspinatus and infraspinatus tendons are known to be responsible for the dorsocranial displacement of this key fragment [1]. In elderly patients with humeral head fractures, concomitant chronic degenerative rotator cuff pathologies are common [3,4]. Milgrom et al., for example, found rotator cuff lesions in 80% of asymptomatic patients 80 years of age and older [5].

Corresponding to the coexistent pattern of rotator cuff degeneration and humeral head fractures, it would be desirable to identify reciprocal influences between these pathologies [6]. The hypothesis was that patients with chronic degenerative changes of the posterosuperior rotator cuff do not show the typical dorsocranial displacement of the greater tuberosity, as described by Neer [1]. The aim of the present study was to evaluate whether the integrity of the posterosuperior rotator cuff influences the displacement characteristics of the greater tuberosity in proximal humeral fractures [7].

## 2. Materials and Methods

**Figure 1:**
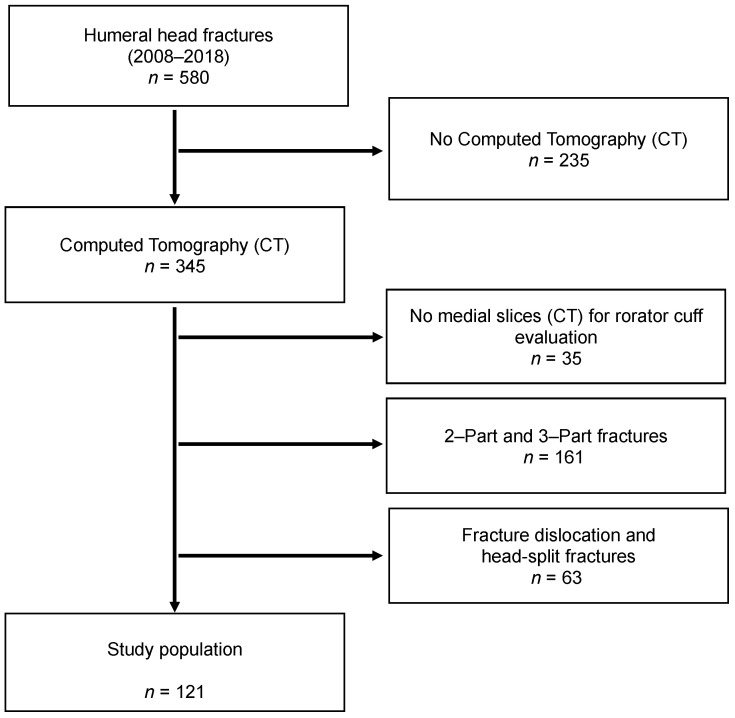
Flowchart of case inclusion/exclusion. The study population consists only of four-Part fractures of the humeral head.

Patients with humeral head fractures diagnosed in our trauma department between 2008 and 2018 were identified. Patients with computed tomography (CT) scans of the affected shoulder were included. CT scans with low image quality or missing medial slices to assess the rotator cuff were excluded. All fractures were categorized according to Neer’s classification system (Table 1) [1]. Two- and Three-Part fractures, as well as fracture-dislocations and head-split fractures, were also excluded in order to generate a homogenous study population of four-Part fractures without fracture-dislocation (Figure 1). The study has been approved by the Ethics Committee at the University of Regensburg (20-1848-104).

### 2.1. Radiological Evaluation

The radiological evaluation was carried out on the basis of CT scans of the affected shoulder. Next to axial slices, parasagittal and coronal reconstructions were used for the radiological measurements. All measurements were performed digitally using the software package OsiriX MD Version 6.5 (Pixmeo, Berne, Switzerland).

### 2.2. Head-Shaft Angle (HSA)

**Figure 2:**
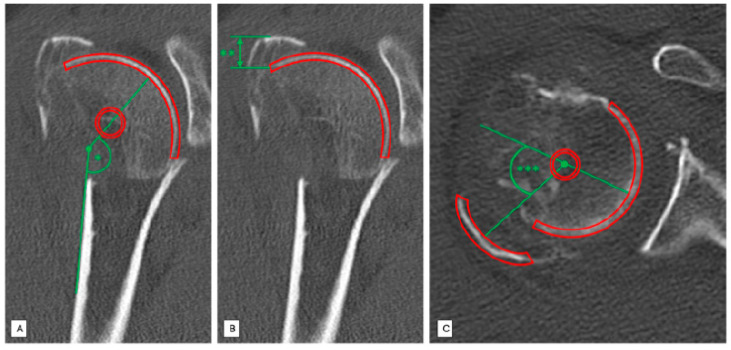
Standardized radiological measurements in a patient with a Neer type 4 fracture injury of the right proximal humerus. (**A**) Standardized radiological measurement (*) of the head-shaft angle [°] in coronal slices. (**B**) Standardized radiological measurement (**) of the greater tuberosity displacement [mm] in vertical plane in coronal slices. (**C**) Standardized radiological measurement (***) of dorsal displacement [°] angle of the greater tuberosity in axial slices.

The head–shaft angle is created by a line parallel to the axis of the humeral shaft and perpendicular to the anatomical neck plane. We measured every head–shaft angle of the 121 included patients in the coronal plane to distinguish between varus (HSA < 125°) and non-varus fractures (HSA > 125°) (Figure 2A).

### 2.3. Position Determination of the Greater Tuberosity in the Vertical Plane (Cranial Displacement)

The displacement of the greater tuberosity in the vertical plane was measured using coronary reconstructions. Firstly, the most distant part of the fragment and the initial attachment of this fragment were identified and the distance between these two was quantified [mm] (Figure 2B).

### 2.4. Position Determination of the Greater Tuberosity in the Horizontal Plane (Dorsal Displacement)

The position of the greater tuberosity in the horizontal plane was evaluated by analyzing axial CT slices. Therefore, determination of dorsal displacement of the greater tuberosity was measured using an angle [°] formed by a line that passes the original insertion of the greater tuberosity and a line through the center of the greater tuberosity fragment (Figure 2C).

### 2.5. Classification of Fatty Degeneration of Rotator Cuff

**Figure 3:**
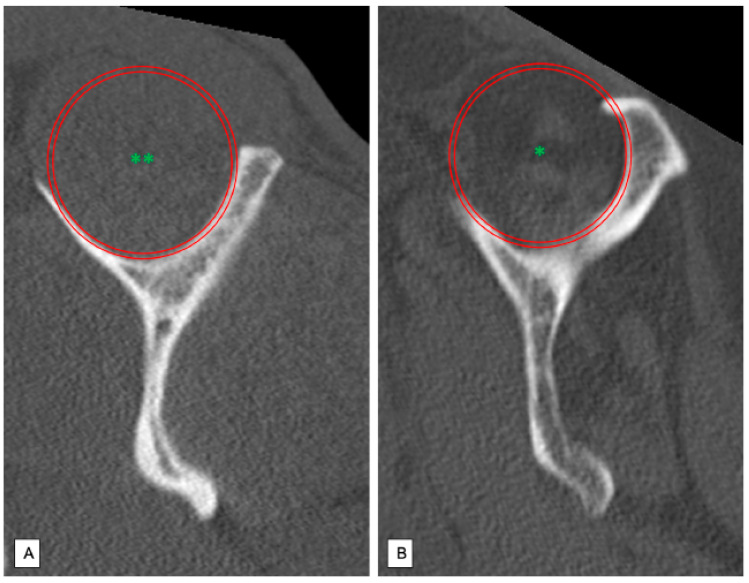
Analysis of the fatty degeneration of the supraspinatus muscle using the Goutallier classification system in parasagittal slices; Red Circle: Supraspinous fossa. (**A**) Goutallier grade 0: No fatty degeneration of the supraspinatus muscle (**). (**B**) Goutallier grade 3–4: Advanced fatty infiltration of the supraspinatus muscle (*).

The posterosuperior rotator cuff was evaluated using the CT-based classification system described by Goutallier [8]. Medial parasagittal slices were set in a typical “Y-shaped-position”. Thus, the supraspinous fossa could be displayed since the plane perpendicular to the scapula runs through the medial border of the coracoid process [9]. Muscular state of the supra- and infraspinatus muscle was then assessed according to Gouttallier’s classification system of fatty degeneration (grade 0 = no fatty infiltration; grade 1 = low fatty infiltration; grade 2 = less muscular fat than muscle mass; grade 3 = fatty degeneration identical with muscle mass; grade 4 = increased fatty degeneration compared to muscle mass). The patients were then divided into two groups: No or minimal fatty infiltration (Goutallier grade 0–1) (Figure 3A) and advanced fatty degeneration of the rotator cuff (Goutallier grade 2–4) (Figure 3B).

### 2.6. Statistical Analysis

Statistical analysis was carried out using SPSS software package version 25 (SPSS Inc., Chicago, IL, USA). The independent t-test was used to compare continuous variables after determining that all variables were normally distributed (Kolmogorov–Smirnov normality test). *p*-values < 0.05 were considered significant. All graphs are displayed with mean value and 95% confidence interval.

## 3. Results

**Figure 4:**
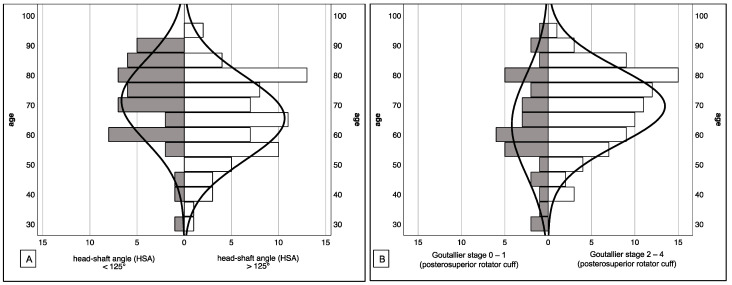
Age distribution of the study collective with regard to the head-shaft angle (**A**) and the posterosuperior rotator cuff (**B**) Patients with varus-displaced fractures and advanced signs of fatty degeneration of the posterosuperior rotator cuff are significantly older compared to the others (*p* ≤ 0.05).

A total of 121 patients (86 female, 35 male) with a mean age of 67.7 years (female Ø71.7 ± 11.9 and male Ø57.9 ± 14.8) met the inclusion/exclusion criteria. Age and sex distribution in our population was normal (*p* < 0.000). 38% (46/121) showed an HSA of less than 125° (mean age 71.4 years), and 62% (75/121) of the patients had an HSA of more than 125° (mean age 65.5 years). In 86 patients (71%; mean age 69.5 years), high-grade fatty degeneration of the posterosuperior rotator cuff was observed (Goutallier 2–4). Statistical analysis revealed that patients with varus displaced humeral head fractures (HSA < 125°) and patients with advanced signs of rotator cuff degeneration (Goutallier 2–4 of the posterosuperior cuff) were older compared to the others (HSA: *p* = 0.026; Fatty degeneration: *p* = 0.027). Figure 4 displays the age distribution of the study collective with regard to the HSA and the fatty degeneration of the posterosuperior rotator cuff.

### 3.1. Cranial Displacement of the Greater Tuberosity

Analyzing the entire collective (*n* = 121) cranial displacement of the greater tuberosity of 4.5 mm ± 3.8 mm (0–15 mm) was observed. No significant difference between the displacement height and the condition of the supraspinatus muscle could be detected (Goutallier 0–1: 4.9 mm + 4.4 mm vs. Goutallier 2–4: 4.3 ± 3.5 mm; *p* = 0.428).

**Figure 5:**
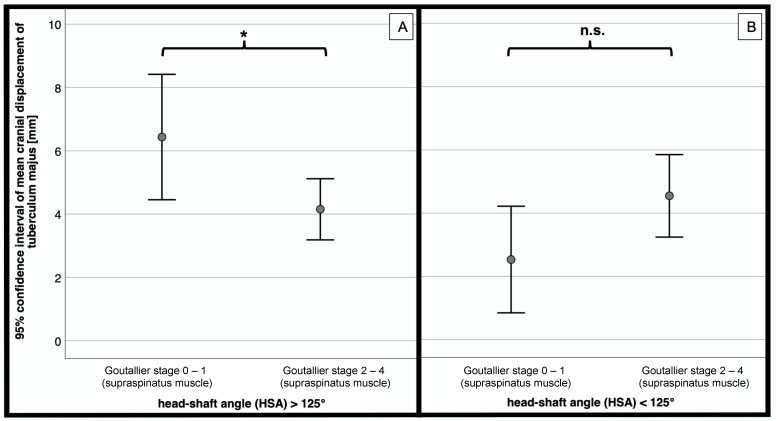
Mean cranial displacement [mm] of greater tuberosity depending on the Goutallier stage of the supraspinatus muscle. (**A**) Head-shaft angle > 125° (*n* = 75), (**B**) head-shaft angle < 125° (*n* = 46). * = significant, n.s. = not significant.

For those fractures with a head–shaft angle of more than 125° a significantly increased cranial displacement of the greater tuberosity was measured in patients with no or minimal signs of supraspinatus muscle degeneration (Goutallier 0–1) compared to those with advanced signs of fatty infiltration (Goutallier 2–4; *p* = 0.020; Figure 5A).

In varus dislocated proximal humeral fractures, the cranial displacement tended to be lower in patients with Goutallier grade 0–1 compared to high-grade degenerated supraspinatus muscles (Goutallier 2–4) without significant differences (*p* = 0.467; Figure 5B).

### 3.2. Dorsal Displacement of the Greater Tuberosity

Within the entire study collective (*n* = 121), a dorsal displacement of the greater tuberosity of 25.8° ± 28.7° was measured. Comparing the dorsal displacement grade in patients with largely intact infraspinatus muscles (Goutallier 0–1) to those with advanced signs of fatty degeneration (Goutallier 2–4), no significant differences could be detected (Goutallier 0–I: 27.3° ± 30.8° vs. Goutallier II–IV: 21.5° ± 21.2°; *p* = 0.332).

**Figure 6:**
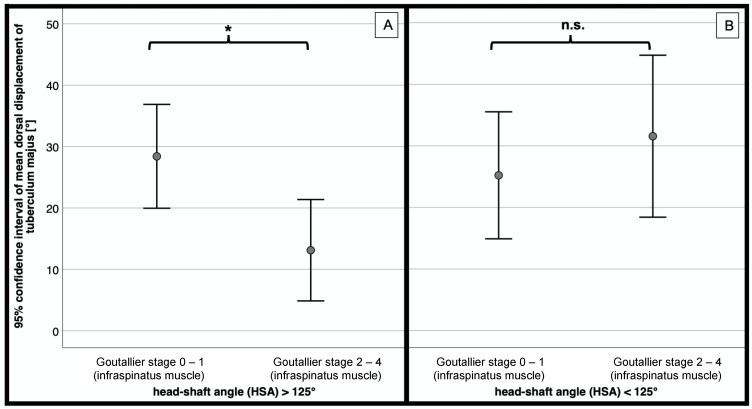
Mean dorsal displacement [°] of greater tuberosity depending on the Goutallier stage of the infraspinatus muscle. (**A**) Head-shaft angle > 125° (*n* = 75). (**B**) head-shaft angle < 125° (*n* = 46). * = significant, n.s. = not significant.

For non-varus displaced fractures with a head-shaft angle of more than 125°, significant less dorsal displacement of the greater tuberosity fragment was noticed for higher grades of infraspinatus muscle degeneration (Goutallier 2–4) compared to patients with less fatty infiltration (Goutallier 0–1; *p* = 0.010; Figure 6A).

Varus displaced fractures in patients with no or minimal signs of fatty infiltration of the infraspinatus muscle (Goutallier 0–1) tended to have less dorsal displacement of the greater tuberosity compared to those with high-grade degenerated infraspinatus muscles (Goutallier 2–4; *p* = 0.467; Figure 6B).

## 4. Discussion

The key findings of the present study are:

− In non-varus four-part humeral head fractures, cranial and dorsal displacement of the greater tuberosity depends on the status of the posterosuperior rotator cuff.
− In varus displaced humeral head fractures, no correlation between displacement of the greater tuberosity and the condition of the posterosuperior rotator cuff could be detected.

In clinical practice, dorsocranial displacement of the greater tuberosity in humeral head fractures is frequently seen. Neer postulated that displacement of this key fragment is caused by traction of the posterosuperior rotator cuff [1]. Although this theory still acts as an explanatory model, this assumption has not yet been sufficiently investigated [10]. Accordingly, there should be differences in the displacement of the greater tuberosity in patients with a sufficient rotator cuff and patients with an insufficient cuff, e.g., caused by rupture.

In the present study, we assessed the condition of the posterosuperior rotator cuff by evaluating the supraspinatus and infraspinatus muscles according to Goutallier on CT scans [8]. Barry et al. showed that the degree of fatty infiltration, especially of the supra- and infraspinatus muscles, are related to the tear severity [11]. This study demonstrated that in the case of an advanced fatty infiltration (Goutallier ≥ 2), only 6.5% had no supraspinatus tendon tears, while the majority of patients suffered from partial or complete ruptures [11].

Overall, demographic change in western countries has led to an increased number of dislocated proximal humeral fractures in elderly patients [12]. These patients often show preexisting rotator cuff tears [4,5,13]. In the present patient population, the incidence of degenerative rotator cuff pathologies in older patients was also higher than in younger patients. In addition, varus displaced humeral head fractures were more frequent in elderly patients, similar to previously published studies [14].

Interestingly, we found differences in the displacement mode of the greater tuberosity in patients with varus displaced fractures compared to others. In fractures with a head–shaft angle of more than 125°, we detected a significantly higher displacement grade of the greater tuberosity in patients without or with minimal degenerative changes of the posterosuperior cuff compared to patients with high-grade degeneration. These results support the theory that the displacement of this fragment is merely caused by force vectors of the cuff, as described by Neer [1].

In varus displaced fractures, we have made contrary observations. In patients with high-grade degeneration of the posterosuperior cuff, the greater tuberosity tended to be more displaced than in patients with inconspicuous rotator cuff status.

A possible explanation could be that in varus impacted fractures, an impression injury caused by the acromion is more likely than fracture displacement due to rotator cuff traction. The intact supra- and infraspinatus tendons can then possibly act as a placeholder in these fractures and thus partially prevent a fragment displacement of the greater tuberosity.

Several limitations of the present study have to be discussed. Only CTs were used to assess the rotator cuff. Although we have only used scans with existing medial slices and computed tomography is a proven diagnostic modality to classify fatty infiltration of the rotator cuff [15,16], some limitations of CT for assessing the rotator cuff status are obvious.

It is also possible that advanced fatty infiltrated rotator cuff parts insert at the greater tuberosity and contribute a part to the displacement by residual activity. In addition, it is possible that acute complete ruptures, without relevant fatty infiltration of the cuff but without any mechanical possibility to contribute to fragment displacement, are included in the study collective. Nevertheless, a fatty degeneration of the musculature is already detectable 6 weeks after a complete rupture, which is why the number of missed complete ruptures should be limited [8,17]. Additionally, this study is a radiological study without evaluation of clinical parameters. Nevertheless, clinical data could not help answering the questions of the present study. One important point is that there is a risk of measurement inaccuracy, although scans with low image quality have been excluded. A certain deviation due to different measurements of the displacement cannot be denied.

Despite the limitations mentioned above, this study is the first to show that in some humeral head fractures, the displacement of the greater tuberosity occurs particularly in intact posterosuperior rotator cuffs, thus supporting Neer’s theory that fragment displacement is due to rotator cuff traction [1]. However, in some fractures (varus displaced fractures), fragment displacement follows a different pathophysiological pattern.

To what impact these results may influence treatment strategies for such injuries, or whether it may be useful as a prognostic tool of such fracture patterns must be clarified in further studies.

## 5. Conclusions

The present study demonstrates that in non-varus four-part humeral head fractures, cranial and dorsal displacement of the greater tuberosity depends on the status of the posterosuperior rotator cuff, whereas in varus displaced humeral head fractures, no correlation between the displacement of the greater tuberosity and the condition of the posterosuperior rotator cuff could be detected. These results imply that the commonly accepted theory of greater tuberosity displacement in humeral head fractures by rotator cuff traction cannot be applied to all fracture types.

## Figures and Tables

**Table 1:** Schematic overview of Neer’s modified classification system [1].

Fracture Type	Number of Fragments		
	2	3	4
I			
II	anatomical neck		
III	surgical neck		
IV	greater tuberosity		
V	lesser tuberosity		
VI	anterior or posterior luxation		

I: minimal dislocation, under 1 cm and less angulation than 45°.

## Data Availability

Data is available.
